# Analysis of Factors Affecting the Diagnostic Efficacy of Frozen Sections for Tumor Spread Through Air Spaces in Lung Adenocarcinoma

**DOI:** 10.3390/cancers17132168

**Published:** 2025-06-27

**Authors:** Xin Liu, Yun Ding, Jie Ren, Jiuzhen Li, Kai Wang, Shuai Sun, Weiran Zhang, Meilin Xu, Yuhao Jing, Guozheng Gao, Wenkang Zong, Daqiang Sun

**Affiliations:** 1 Clinical School of Thoracic, Tianjin Medical University, Tianjin 300070, China; dr_lllllx@163.com (X.L.); dymail94@163.com (Y.D.); jie2012kl@sohu.com (J.R.); datanju123456@163.com (J.L.); nickwang1996@163.com (K.W.); jimmywhitelying@gmail.com (S.S.); bottomchest@sina.com (W.Z.); meilinxugh@163.com (M.X.); time2.17chest@sina.com (G.G.); zwkyouxiang@163.com (W.Z.); 2 Department of Thoracic Surgery, Tianjin Jinnan Hospital, Tianjin 300350, China; 3 Department of Thoracic Surgery, Tianjin Chest Hospital, Tianjin 300222, China; 4 Department of Pathology, Tianjin Chest Hospital, Tianjin 300222, China; 5 Clinical School of Thoracic, Tianjin University, Tianjin 300072, China; jingyuhao0001@163.com

**Keywords:** tumor spread through air spaces, adenocarcinoma, non-small-cell lung cancer, frozen sections, diagnostic efficacy

## Abstract

**Objectives**: This study aimed to determine the factors affecting the diagnostic efficacy of frozen sections for assessing tumor spread through air spaces (STAS) and to provide suggestions for the sampling of these frozen sections. **Methods**: Cases of invasive adenocarcinoma with a pathological diagnosis of stage IA-IIIB were screened, and frozen and paraffin sections were reviewed. Using paraffin sections as the gold standard, the consistency of frozen pathological diagnosis of STAS was calculated. Factors that may affect STAS diagnosis in frozen sections were screened, and a nomogram was drawn. **Results**: The sensitivity of frozen sections in STAS diagnosis was 55.4% (108/195), the specificity was 74.5% (254/321), and the kappa value was 0.35. In the subsequent logistic regression, the ratio of the tissue diameters of the frozen and paraffin sections, number of frozen section sheets, clarity of the tumor boundary, and number of alveoli from the peritumoral area to tissue edge were all statistically significantly significant (*p* < 0.05) factors affecting the frozen STAS diagnostic efficacy. **Conclusions**: The diagnostic efficacy of frozen sections for STAS is poor. In our study, the tissue diameter ratio of the frozen to paraffin sections, the number of frozen sections, the clarity of the tumor boundary, and the number of alveoli from the peritumoral tissue to the tissue edge were considered independent factors affecting diagnostic consistency. The accuracy of the frozen section analysis in STAS diagnosis can be improved by our reasonable suggestions on frozen sampling, making it a reliable indicator of the surgical method.

## Simple Summary

Accurate diagnosis during lung cancer surgery is essential for guiding treatment decisions. Surgeons often rely on a rapid method called frozen section analysis to assess how a tumor has spread inside the lungs. One pattern of spread, known as tumor spread through air spaces, can be difficult to detect accurately using this method. In this study, we reviewed a large number of lung cancer cases to evaluate how well frozen section analysis can identify this pattern and what factors influence its accuracy. We found that certain features of the tumor may lead to inconsistent results. Our findings highlight the need for caution when relying solely on frozen section tests and suggest ways to improve diagnostic practices. This research can help doctors make better decisions during surgery and improve outcomes for patients with lung cancer.

## 1. Introduction

Lung cancer is the leading cause of cancer-related death worldwide [1]. At present, the incidence of lung adenocarcinoma is increasing, and it has surpassed squamous cell carcinoma to become the main pathological type of non-small cell carcinoma. While surgery remains an important treatment option for invasive lung adenocarcinomas, postoperative recurrence is a serious issue during treatment for invasive lung adenocarcinoma and even non-small cell carcinoma. Despite years of research, factors such as pathological grade, vasculature, and pleural invasion remain associated with postoperative recurrence [2], which occurs even among patients who are not exposed to these factors. Recently, the term spread through air spaces (STAS) has been widely used. In 2015, the World Health Organization (WHO) formally introduced this concept in its new classification of lung cancer [3]; it was defined as the spread of micropapillary clusters, solid nests, and/or individual cancer cells into the alveolar lumen beyond the margin of the main tumor in the alveolar cavity of the lung. STAS is a histological feature with prognostic significance, and the occurrence of STAS indicates worse recurrence-free survival (RFS) and overall survival (OS) [4]. At the same time, segmentectomy in patients who were STAS-positive has been associated with worse RFS and OS than lobectomy [5,6,7,8,9]. Therefore, the early identification of STAS status is very important for the formulation of a surgical plan. Intraoperative frozen pathology can quickly provide information on pathological tumor characteristics and guide the choice of surgical methods [8,9,10,11]. Unfortunately, the diagnostic efficacy of frozen STAS remains poor at present, and its overall sensitivity is only 44–54% [7,8,9,12,13], making application in clinical practice difficult. Therefore, this study aimed to identify the factors influencing the accuracy of intraoperative frozen pathology, attempting to suggest a sampling method for frozen sections to improve the sensitivity and specificity of frozen section STAS diagnosis. This would help make frozen STAS diagnosis as reliable an indicator as possible to serve as a basis for clinical decision-making, leading to improved patient survival. However, the intraoperative diagnosis of STAS using frozen sections remains suboptimal, with poor sensitivity and inconsistent accuracy across studies. The specific reasons for this diagnostic limitation and potential strategies for improvement have not been fully elucidated. Therefore, we aim to bridge this gap in this study.

## 2. Materials and Methods

### 2.1. Study Population

The relevant data of patients who underwent lung cancer surgery in the Department of Thoracic Surgery of Tianjin Chest Hospital and the Department of Thoracic Surgery of Tianjin Jinnan Hospital from January 2015 to December 2019 were retrospectively collected. The inclusion criteria were as follows: (1) non-metastatic invasive adenocarcinoma confirmed by postoperative pathology, (2) complete R0 resection, and (3) pathological diagnosis of stage IA-IIIB. The exclusion criteria were as follows: (1) pre-operative neoadjuvant therapy, (2) consideration of multiple primary tumors in postoperative pathology, and (3) no intraoperative frozen pathological examination. This study was approved by the Ethics Committee of Tianjin Chest Hospital and the Ethics Committee of Tianjin Jinnan Hospital, which waived the requirement for informed consent due to the retrospective nature of the study.

### 2.2. Specimen Processing and Baseline Data Collection

Patient age, sex, surgical procedure, pathological stage, and T stage were recorded. For each case, a section was cut from a fresh tumor. Frozen sections were typically taken from the interface between the tumor and peritumoral lung, aiming to capture the transition zone. Sampling was guided by gross morphology to ensure the inclusion of alveolar spaces for potential STAS detection, and each sample was embedded and frozen in optimal cutting temperature (OCT) compounds and cut into 4 μm sections on positively charged slides for hematoxylin and eosin (H&E) staining to obtain 4 μm thick frozen sections. The remaining specimens that were not used for the frozen sections were prepared as paraffin sections according to the conventional section-making process. The diagnostic consistency of STAS in frozen sections was compared to that in paraffin sections.

### 2.3. Records of Pathological Indicators

**Figure 1:**
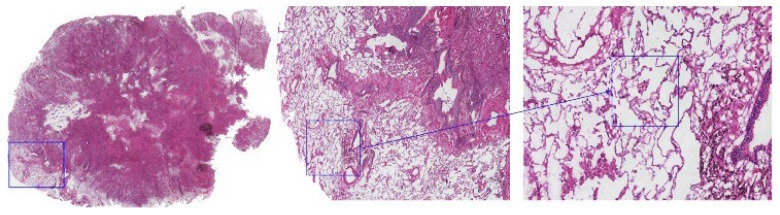
Micropapillary clusters were observed in the air space of the lung beyond the margin of the main tumor under a digital biopsy scanner and were recorded as STAS-positive. The three panels represent progressively increasing magnifications—1× (**left**), 100× (**middle**), and 400× (**right**)—acquired using a digital scanner. The blue square in the low-power image indicates the region selected for high-power observation. The magnified image was captured at 400× total magnification (objective lens 40×, eyepiece 10×).

**Figure 2:**
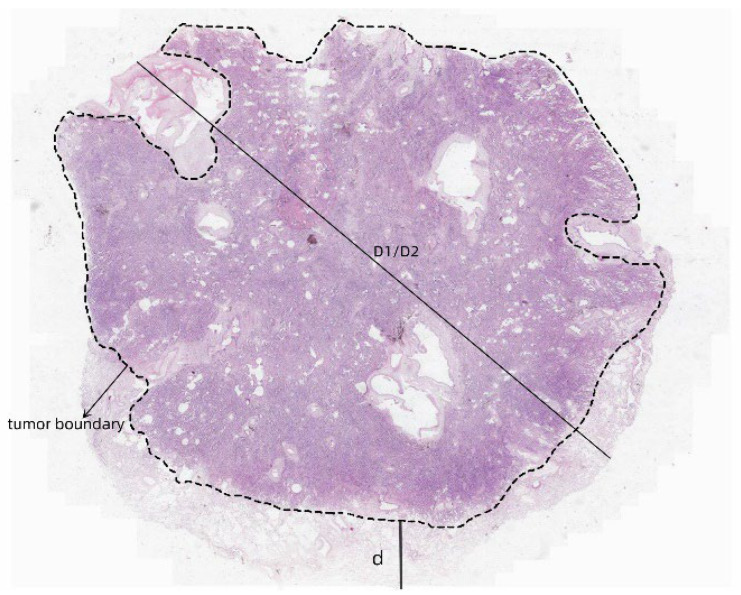
Examples of tumor boundaries and d, D1, and D2 under frozen and paraffin sections. D1 indicates the distance from the tumor edge to the closest point of alveolar spread, while D2 refers to the distance to the furthest point. d represents the minimum distance from the tumor to the outer edge of the specimen. The dotted line outlines the tumor boundary. The square area image was captured at 1× magnification using a digital scanner.

**Figure 3:**
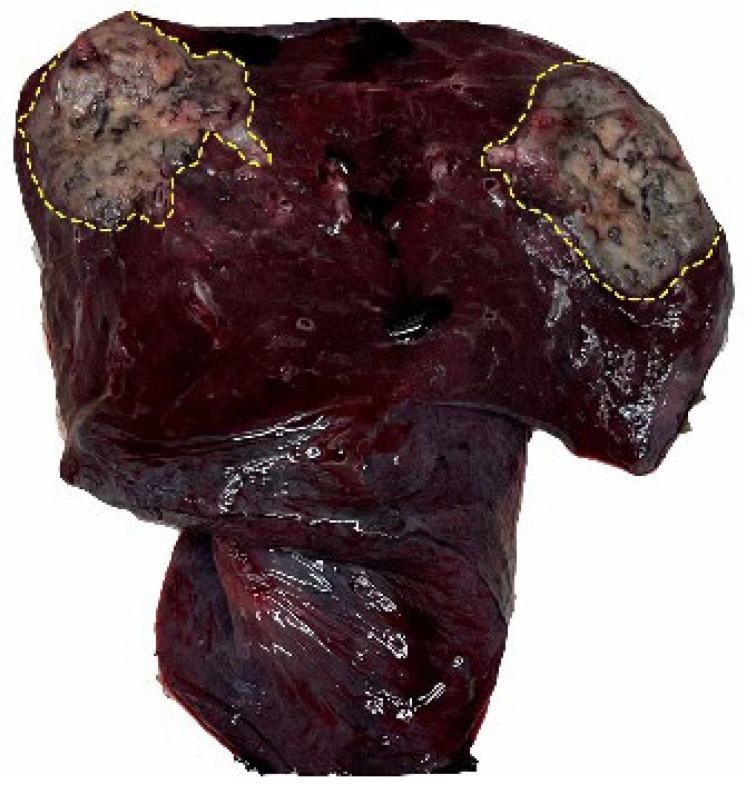
The boundary of the tumor tissue is clear when viewed by the naked eye. The dotted yellow line indicates the outline of the tumor boundary.

In this study, each pathological section was evaluated by three experienced pathologists. All frozen and paraffin-embedded sections were evaluated for each patient. The presence or absence of STAS was recorded in each section. STAS was defined as described in the 2015 WHO classification of thoracic neoplasms: clusters of micropapillae, solid nests, and/or individual cancer cells spreading into the air gap of the lung parenchyma beyond the margin of the main tumor (as shown in Figure 1). Artifacts were defined as linear cell strips stripped from the alveolar wall and clusters of tumor cells distributed randomly and discontinuously in the tissue. The following indicators were recorded, measured, and calculated: the diameter of the tissue under the paraffin section (D1, cm) and the diameter of the tissue under the frozen section (D2, cm). For irregular specimens, both the longest and shortest axes were measured and averaged. Digital scanning software (Version 3.2.1) with standardized calibration was used to measure the distance between the tumor boundary and the tissue edge under the digital biopsy scanner (d, μm). The shortest perpendicular distance from the visible tumor edge to the tissue margin was recorded. In curved margins, linear approximation was applied. All measurements were taken by two observers and averaged, as shown in Figure 2 and Figure 3 for the D2/D1 ratio and d/D2 ratio. The clarity of the tumor boundary under the naked eye was assessed macroscopically by pathologists based on whether the margin between the tumor and surrounding normal lung tissue could be clearly delineated by the naked eye. A “clear” boundary referred to a sharply demarcated edge resembling a continuous line, while an “unclear” boundary indicated a gradual, indistinct transition suggestive of tumor infiltration. All evaluations were conducted jointly by three experienced thoracic pathologists with consensus resolution to minimize observer bias. The number of alveoli from the outer edge of the specimen under the digital section scanner was divided into two groups: 5–10 alveoli and >10 alveoli. The number of alveoli at the peripheral edge was counted under low-power magnification (×40), with the most representative field adjacent to the tumor selected for assessment. Although the grouping threshold (5–10 alveoli vs. >10 alveoli) was not derived from a formal ROC analysis or the specific prior literature, it was empirically determined based on preliminary case reviews. This approach was found to be practical and reproducible. Future studies may further refine this cutoff using quantitative analysis or multi-center validation. For frozen sections, if no normal alveolar tissue beyond the tumor boundary was observed under the microscope, STAS was recorded as “not observed”. The corresponding d and alveolar numbers were recorded as 0, and the sections were included in the frozen STAS unevaluable group. D1 and D2 were averaged according to the number of sections. The clarity of the tumor boundary was assessed macroscopically by pathologists and categorized as “clear” or “unclear” based on whether the margin between the tumor and adjacent normal tissue could be clearly delineated by the naked eye. For quality control, three pathologists independently reviewed 50 randomly selected cases, and interobserver agreement was measured using the kappa statistic.

### 2.4. Statistical Methods

Statistical analyses were conducted using IBM SPSS Statistics for Windows, version 26.0 (IBM Corp., Armonk, NY, USA). Continuous variables with a normal distribution are presented as mean ± standard deviation. Enumeration data are expressed as percentages (%), and comparisons between groups were performed using the χ^2^ test or Fisher’s exact test. The kappa test (K) was used for consistency testing. According to the guide, kappa values were interpreted as follows: <0.4, low agreement; 0.4 to <0.6, moderate agreement; 0.6 to <0.8, substantial agreement; and 0.8 to <1.0, almost perfect agreement. The results from the paraffin sections were used as the gold standard. Factors that may affect the diagnostic efficacy of STAS were screened using univariate logistic regression, and statistically significant factors in this analysis were included in the multivariate logistic regression analysis. Finally, the independent factors influencing the diagnostic consistency of STAS between frozen and paraffin-embedded sections were obtained. Statistical significance was set at *p* < 0.05. The rms package of R 4.2.0 was used to draw a nomogram for STAS diagnostic consistency.

## 3. Results

### 3.1. Baseline Characteristic Data and Diagnostic Efficacy of Frozen Sections for Invasive Adenocarcinoma

Among 649 patients included in this study, the incidence of STAS in paraffin sections was 38.2% (248/649). Among them, 133 patients had no normal lung tissue around the tumor tissue in the intraoperative frozen section. The remaining 516 patients were evaluated for the diagnostic consistency of STAS in frozen sections and paraffin sections. In the excluded cohort of 133 patients, the accuracy of frozen pathology for the diagnosis of pulmonary malignancy was 95.5% (127/133). This difference was not statistically significant (*p* = 0.872), as shown in Table 1. The frozen section containing normal lung tissue at the tumor boundary could also ensure the accuracy of intraoperative freezing for the diagnosis of lung cancer. Excluding the 133 patients in the non-evaluable group, the baseline characteristics data of the study cohort comprising 516 patients are shown in Table 2.

### 3.2. Efficacy of Frozen Section STAS Diagnosis

In paraffin sections, the incidence of STAS was 37.8% (195/516), whereas the positive rate for STAS in frozen sections was 33.9% (175/516). Among the 195 cases of STAS in the paraffin sections, 108 were also positive for STAS in the frozen sections (sensitivity, 55.4%). Of the 321 cases with no STAS in the paraffin sections, 254 also had no STAS in the frozen sections (specificity, 74.5%). Among the 175 cases with STAS in the frozen sections, 108 cases (positive predictive value [PPV], 61.7%) had STAS in the paraffin sections. Of the 341 cases without STAS in the frozen sections, 254 (negative predictive value [NPV], 74.5%) also had no STAS in the paraffin sections. Compared to the paraffin sections, the kappa value of frozen sections for STAS diagnosis was 0.35, which was consistently low, as shown in Table 3.

### 3.3. Logistic Regression for Screening Factors Affecting the Diagnosis of Frozen STAS

**Figure 4:**
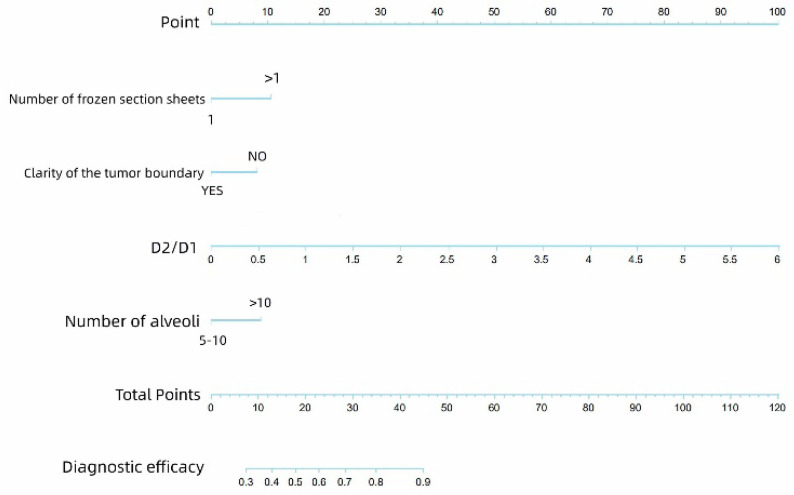
The nomogram shows how the D2/D1 ratio, number of alveoli from the outer edge of the specimen under the digital section scanner, number of alveoli, and clarity of the tumor boundary affect the diagnostic efficacy of STAS.

Univariate analysis showed that the D2/D1 ratio, number of alveoli from the outer edge of the specimen under the digital section scanner, number of alveoli, diameter of tissue under paraffin section (D1, cm), and clarity of the tumor boundary were statistically significant (*p* < 0.05), as shown in Table 4. In the subsequent multivariate logistic regression, the D2/D1 ratio, number of alveoli from the outer edge of the specimen under digital section scanner, number of alveoli, and clarity of the tumor boundary were statistically significant (*p* < 0.05), as shown in Table 5; these factors were considered independent factors affecting the consistency of frozen STAS diagnosis. The results are visually illustrated in the nomogram shown in Figure 4.

## 4. Discussion

STAS is defined as the protrusion of lung cancer cells into the lung parenchyma beyond the outer edge of the main tumor. In other words, tumor cells appear in the air spaces outside the tumor tissue. In a study by Walts et al. [14], the prevalence of STAS was 95.8%. In other reports, the prevalence of STAS ranges from 14.8% to 51.4%, whereas the prevalence of STAS in this study was 47.9%. We believe that this may be due to the different clinical stages of the patients included in the study. Specifically, the cohort in the study by Walts et al. may have included a disproportionately high number of advanced-stage or poorly differentiated tumors, leading to increased STAS visibility. Our study population included a wider spectrum of stages, primarily early-stage adenocarcinoma.

The assessment of STAS status depends on the acquisition of tissue specimens; however, according to previous studies, invasive pre-operative methods, including computed tomography (CT)-guided lung biopsy and tracheoscopy, failed to obtain sufficient samples. Hence, it is difficult to assess STAS status. Therefore, intraoperative frozen pathology has high diagnostic potential in the evaluation of STAS.

Although it is inconclusive whether patients with a positive STAS status determined by frozen pathology should undergo lobectomy, a consensus regarding STAS as a prognostic factor has been reached in a series of previous studies: The presence of STAS means that patients have worse RFS and OS [4,7,8,9,15]. To reduce the recurrence of invasive adenocarcinoma after surgery, the STAS status should be clarified before and during surgery. The accuracy of frozen pathology in the diagnosis of STAS has shown varying results in different studies. In a study by Zhou et al. [16], the sensitivity of STAS in frozen sections was 55%, the PPV was 48%, the specificity was 80%, the NPV was 85%, and the accuracy was 74%. In a study by Villalba et al. [17], frozen pathology showed low sensitivity (44%), high specificity (91%), and relatively high accuracy (77%) for STAS. In our study, compared to the paraffin sections, the sensitivity of STAS in the frozen sections was 55.4%, the PPV was 61.7%, the specificity was 79.1%, the NPV was 74.5%, and the accuracy was 70.2%. The kappa value was 0.35, indicating a low consistency. These results are similar to those of previous studies [15,16], suggesting that although frozen pathology has the potential to evaluate STAS, the diagnostic efficiency is not high at present and needs further improvement.

Based on the findings so far, we believe that there are multiple reasons for this poor consistency. The first is the existence of artifacts. Spread through a knife surface (STAKS) refers to a process secondary to lung tissue dissection that causes the artificial displacement of tumor cells by knife manipulation along the section plane and processing. Merovic et al. verified the occurrence of the STAKS phenomenon caused by cutting the lung tissue with a knife [18]. Xie et al. [19] also showed that the occurrence of single-celled STAS is related to the STAKS phenomenon. However, as ours was a retrospective study, artifacts generated by STAKS could not be avoided, and further prospective studies are required to exclude STAKS-generated artifacts. Some morphological clues, such as the linear alignment of tumor cells along the cutting plane or isolated cell clusters lacking alveolar structural landmarks, may suggest STAKS rather than true STAS. To reduce the risk of artifact generation, procedural precautions—such as using sharp blades, minimizing mechanical compression, and performing immediate freezing post-excision—should be implemented during frozen section preparation. As a second reason, some alveoli were poorly inflated. Owing to the extrusion operation during surgery and the transport of pathological specimens, some alveolar tissues were poorly inflated, which affected the diagnosis of frozen pathology and the evaluation of frozen STAS [20,21,22]. Morimoto et al. [23], in a recent multi-center study, further confirmed that optimized sampling during frozen section examination can improve the specificity of STAS detection without sacrificing sensitivity. The authors of [24] confirmed that the insufficient expansion of alveolar cavities in frozen sections affects the microscopic observation of tumor cell clusters and subsequently affects the diagnosis of STAS in frozen sections. Jae et al. [21] also noted that poorly inflated alveolar cavities could interfere with the diagnosis of frozen sections and proposed inflating frozen specimens, which allows the intact alveolar morphology to be extended. This approach facilitates the diagnosis of minimal lesions (e.g., atypical adenomatous hyperplasia and bronchioloalveolar carcinoma). Whether inflating the specimen by puncturing the pleura with a fine needle increases the spread of tumor cells remains to be determined. However, the proposal of this treatment method provides a novel idea for solving the problem of specimens being poorly inflated. The third reason for poor consistency is dyeing and sampling issues. In the process of reviewing previous frozen sections, we noticed that some sections had slight discoloration. Even if multiple pathologists were involved in this study for joint evaluation, it would be difficult to avoid the occurrence of missed diagnoses. The premise of intraoperative frozen pathology involves being as rapid and accurate as possible. Therefore, when taking frozen pathological samples, experienced sampling physicians tend to select the area with the most obvious lesions under the macroscopic state to prepare frozen sections to facilitate the diagnosis of benign and malignant specimens, resulting in a lower amount of surrounding normal lung tissue and making it difficult to evaluate STAS in frozen sections. In parallel, several CT-based radiomics and deep learning models have exhibited promising performance for the non-invasive, pre-operative prediction of STAS [25,26,27,28].

We believe that these errors can be reduced by improving the preparation of sections; therefore, relevant indicators were selected and included in our study. Our results show that the tissue diameter ratio of the frozen to paraffin sections, the number of frozen sections, the clarity of the tumor boundary, and the number of alveoli from peritumoral to tissue edge could affect the consistency of STAS diagnosis between frozen and paraffin sections. Based on these results, we drew a nomogram to provide suggestions for the preparation of frozen sections. The nomogram derived from our multivariate logistic regression allows surgeons and pathologists to estimate the likelihood of consistency between STAS diagnoses using frozen and paraffin sections. This tool may aid intraoperative decision-making by identifying cases in which frozen section results are more likely to be reliable, thereby guiding the extent of surgical resection (e.g., lobectomy vs. segmentectomy).

The tissue diameter ratio of the frozen to paraffin sections should be as large as possible, and the number of alveoli around the cancer to the edge of the tissue should be >10. When these two indicators are not met, increasing the number of frozen sections is recommended to improve the accuracy of STAS diagnosis. However, in determining whether the tumor boundary was clear, we concluded that an unclear border was conducive to STAS diagnosis (*p* < 0.05). We considered that the heterogeneity of the tumor was caused by different clinicopathological features. For example, in a pulmonary nodule showing ground glass opacity (GGO) on CT, the GGO profile usually is gray, soft in texture, small in scope, and unclear in boundary, whereas the profile of partial solid tumors is gray, tough or even hard in texture, large in scope, and clear in boundary. Therefore, to ensure the accuracy of frozen pathology, the lesion area with unclear boundaries and a large sampling area tends to contain more normal lung tissue, which facilitates the detection of STAS clusters, since these parameters make it easier to evaluate STAS in frozen sections. However, tumors with more solid components have clearer profile boundaries. Due to the limitation of the size of frozen sections, especially for large tumors, sampling physicians often choose the most obvious lesion area under the naked eye for the preparation of frozen sections according to their experience. Such areas often exist near the center of the tumor; therefore, they contain less surrounding normal lung tissue, making frozen STAS evaluation difficult. In the process of reviewing the previous frozen sections, we found that tumor tissues with better profile boundaries contained less normal lung tissue. Therefore, to evaluate STAS, including as much peritumoral lung tissue as possible is recommended during the sampling of lesions with clear boundaries. Our findings also show that such a sampling method had no significant effect on the judgment of the nature of the lesion.

This study had some limitations. First, it was a retrospective study. There was no unified standard for frozen pathological sampling, and many factors may have affected the results. Although we adopted numerous methods, bias was unavoidable. Second, the practicability of the consistency prediction model for frozen STAS constructed in this study requires further prospective evaluation. Finally, although multiple pathologists participated in the evaluation, most centers lack pathologists skilled in diagnosing lung tumors. In the process of diagnosing frozen STAS, even if the aforementioned recommendations are achieved, missed diagnoses and misdiagnoses are still possible. Hence, we look forward to the development of objective and reliable technology (such as artificial intelligence technology) to help pathologists diagnose intraoperative frozen STAS, further enhance the sensitivity and specificity of frozen pathological sections for STAS detection, and improve intraoperative decision-making, ultimately improving patient survival. Specifically, deep learning-based image analysis may aid in identifying subtle STAS features, distinguishing them from artifacts, and quantifying diagnostic uncertainty. By learning from large annotated datasets, AI tools could provide real-time decision support to enhance intraoperative accuracy and reproducibility.

In conclusion, the diagnostic efficacy of frozen STAS currently remains poor. Through our study, the tissue diameter ratio of the frozen section to the paraffin section, the number of frozen sections, whether the tumor boundary is clear, and the number of alveoli from peritumoral to tissue edge were determined to affect the diagnostic accuracy of frozen STAS. Therefore, in the process of preparing frozen sections, we should fully consider these factors to improve the diagnostic efficacy of STAS. Emerging algorithms such as STASNet and up-to-date CT meta-analyses further underline the clinical potential of imaging-driven STAS assessment [29,30].

## 5. Conclusions

In this study, we screened out factors such as the tissue diameter ratio of frozen to paraffin sections, number of frozen sections, clarity of the tumor boundary, and number of alveoli from the peritumoral tissue to the tissue edge as independent factors affecting the diagnostic efficacy of frozen sections for STAS, and we visually presented them through a nomogram. We also proposed reasonable suggestions related to frozen sampling to improve the accuracy of frozen sections in STAS diagnosis to make it a reliable indicator of the surgical method.

## Figures and Tables

**Table 1:** There was no significant difference in the diagnosis of benign and malignant tumors between the evaluable group and non-evaluable group assessed using paraffin sections.

		Benign	Malignant	Total	The Diagnostic Accuracy of Benign and Malignant
Evaluability of STAS in paraffin sections	No	6	127	133	Non-evaluable group 95.5% vs. evaluable group 95.2%
	Yes	25	491	516	*p* value = 0.872
Total		31	618	649	

**Table 2:** Summary of clinicopathologic data.

Gender, *n* (%)	Male	246 (47.70)
	Female	270 (52.30)
Age (years)	Mean	62.16
	Median	63
	Standard deviation	8.04
	Range	18–80
Frozen sections, *n* (%)	Benign	29 (5.60)
	Malignant	487 (94.40)
Type of surgery, *n* (%)	Lobectomy	489 (94.80)
	Segmentectomy	27 (5.20)
Number of frozen sections, *n* (%)	1	406 (78.70)
	1+	110 (21.30)
Tumor boundary, *n* (%)	Not clear	167 (32.40)
	Clear	349 (67.60)
Diameter of paraffin section, D1 (cm)	Mean	2.19
	Standard deviation	0.88
Diameter of frozen section, D2 (cm)	Mean	2.12
	Standard deviation	0.47
Distance between the tumor boundary and the tissue edge under the digital biopsy scanner (μm), d	Mean	2285.19
	Standard deviation	543.44
Number of alveoli from the outer edge of the specimen under digital section scanner, *n* (%)	5–10	60 (11.60)
	10+	456 (88.40)
Pathological stage, *n* (%)	IA	259 (50.20)
	IB	184 (35.70)
	IIA	2 (0.40)
	IIB	29 (5.60)
	IIIA	39 (7.60)
	IIIB	3 (0.60)
T stage, *n* (%)	1	282 (54.70)
	2	214 (41.50)
	3	16 (3.10)
	4	4 (0.80)
STAS on frozen sections, *n* (%)	No	341 (66.10)
	Yes	175 (33.90)
STAS on paraffin sections, *n* (%)	No	321 (62.20)
	Yes	195 (37.80)

**Table 3:** Compared with paraffin sections, the frozen sections had higher specificity and lower sensitivity for the diagnosis of STAS.

		Paraffin Section		Total	Diagnostic Test Performance of STAS on Frozen Sections
Frozen section		STAS−	STAS+		Sensitivity = 55.4%, NPV = 74.5%
	STAS−	254	87	341	Specificity = 79.1%, PPV = 61.7%
	STAS+	67	108	175	Accuracy = 70.2%
Total		321	195	516	k = 0.35

**Table 4:** Univariate analysis.

	Univariate Analysis		
	*p* Value	HR	95% Confidence Interval
Gender	0.145	0.755	0.517–1.102
Age	0.156	0.983	0.959–1.007
Type of surgery	0.98	0.989	0.423–2.311
Number of frozen sections	0.00	3.025	1.713–5.341
Tumor boundary	0.00	0.218	0.130–0.365
Diameter of paraffin section, D1	0.00	0.567	0.453–0.709
Diameter of frozen section, D2	0.222	1.282	0.860–1.192
D2/D1	0.00	7.283	3.813–13.913
Number of alveoli	0.001	2.477	1.434–4.279
d	0.918	1	1.000–1.000
d/D2	0.931	0.816	0.008–83.141
Pathological stage	0.068		
T stage	0.422	0.88	0.645–1.202

**Table 5:** Multivariate analysis.

	Multivariate Analysis		
	*p* Value	HR	95% Confidence Interval
Number of frozen sections	0.004	2.381	1.314–4.317
Clarity of tumor boundary	0.041	0.503	0.260–0.972
Diameter of paraffin section, D1	0.483	1.171	0.753–1.820
D2/D1 ratio	0.002	3.697	1.630–8.389
Number of alveoli	0.016	2.034	1.143–3.618

## Data Availability

All the relevant data are within the manuscript.

## Abbreviations

WHO	World Health Organization
CT	Computed tomography
RFS	Recurrence-free survival
OS	Overall survival
OCT	Optimal cutting temperature
STAS	Tumor spread through air spaces
H&E	Hematoxylin and eosin
STAKS	Spread through a knife surface
CT	Computed tomography
GGO	Ground glass opacity

## References

[1] Sung H., Ferlay J., Siegel R.L., Laversanne M., Soerjomataram I., Jemal A., Bray F. (2021). Global Cancer Statistics 2020: GLOBOCAN Estimates of Incidence and Mortality Worldwide for 36 Cancers in 185 Countries. CA Cancer J. Clin..

[2] De Giglio A., Di Federico A., Gelsomino F., Ardizzoni A. (2021). Prognostic relevance of pleural invasion for resected NSCLC patients undergoing adjuvant treatments: A propensity score-matched analysis of SEER database. Lung Cancer.

[3] Moreira A.L., Ocampo P.S.S., Xia Y., Zhong H., Russell P.A., Minami Y., Cooper W.A., Yoshida A., Bubendorf L., Papotti M. (2020). A Grading System for Invasive Pulmonary Adenocarcinoma: A Proposal from the International Association for the Study of Lung Cancer Pathology Committee. J. Thorac. Oncol..

[4] Yi E., Bae M.K., Cho S., Chung J.H., Jheon S., Kim K. (2018). Pathological prognostic factors of recurrence in early stage lung adenocarcinoma. ANZ J. Surg..

[5] Travis W.D., Brambilla E., Burke A.P., Marx A., Nicholson A.G. (2015). Introduction to The 2015 World Health Organization Classification of Tumors of the Lung, Pleura, Thymus, and Heart. J. Thorac. Oncol..

[6] Warth A., Beasley M.B., Mino-Kenudson M. (2017). Breaking New Ground: The Evolving Concept of Spread through Air Spaces (STAS). J. Thorac. Oncol..

[7] Kadota K., Nitadori J.I., Sima C.S., Ujiie H., Rizk N.P., Jones D.R., Adusumilli P.S., Travis W.D. (2015). Tumor Spread through Air Spaces is an Important Pattern of Invasion and Impacts the Frequency and Location of Recurrences after Limited Resection for Small Stage I Lung Adenocarcinomas. J. Thorac. Oncol..

[8] Ding Y., Chen Y., Wen H., Li J., Chen J., Xu M., Geng H., You L., Pan X., Sun D. (2022). Pretreatment prediction of tumour spread through air spaces in clinical stage I non-small-cell lung cancer. Eur. J. Cardio-Thorac. Surg..

[9] Toyokawa G., Yamada Y., Tagawa T., Oda Y. (2018). Significance of spread through air spaces in early-stage lung adenocarcinomas undergoing limited resection. Thorac. Cancer.

[10] Yang L., Yang Y., Ma P., Zheng B., Liu W., Zhang Z., Ding N., Liu L., Mao Y., Lv N. (2018). Spread through air spaces predicts a worse survival in patients with stage I adenocarcinomas > 2 cm after radical lobectomy. J. Thorac. Dis..

[11] Kadota K., Kushida Y., Kagawa S., Ishikawa R., Ibuki E., Inoue K., Go T., Yokomise H., Ishii T., Kadowaki N. (2019). Limited Resection Is Associated with a Higher Risk of Locoregional Recurrence than Lobectomy in Stage I Lung Adenocarcinoma With Tumor Spread Through Air Spaces. Am. J. Surg. Pathol..

[12] Masai K., Sakurai H., Sukeda A., Suzuki S., Asakura K., Nakagawa K., Asamura H., Watanabe S.I., Motoi N., Hiraoka N. (2017). Prognostic Impact of Margin Distance and Tumor Spread Through Air Spaces in Limited Resection for Primary Lung Cancer. J. Thorac. Oncol..

[13] Dai C., Xie H., Su H., She Y., Zhu E., Fan Z., Zhou F., Ren Y., Xie D., Zheng H. (2017). Tumor Spread through Air Spaces Affects the Recurrence and Overall Survival in Patients with Lung Adenocarcinoma > 2 to 3 cm. J. Thorac. Oncol..

[14] Walts A.E., Marchevsky A.M. (2018). Current Evidence Does Not Warrant Frozen Section Evaluation for the Presence of Tumor Spread Through Alveolar Spaces. Arch. Pathol. Lab. Med..

[15] Eguchi T., Kameda K., Lu S., Bott M.J., Tan K.S., Montecalvo J., Chang J.C., Rekhtman N., Jones D.R., Travis W.D. (2019). Lobectomy Is Associated with Better Outcomes than Sublobar Resection in Spread through Air Spaces (STAS)-Positive T1 Lung Adenocarcinoma: A Propensity Score-Matched Analysis. J. Thorac. Oncol..

[16] Zhou F., Villalba J.A., Sayo T.M.S., Narula N., Pass H., Mino-Kenudson M., Moreira A.L. (2022). Assessment of the feasibility of frozen sections for the detection of spread through air spaces (STAS) in pulmonary adenocarcinoma. Mod. Pathol..

[17] Villalba J.A., Shih A.R., Sayo T.M.S., Kunitoki K., Hung Y.P., Ly A., Kem M., Hariri L.P., Muniappan A., Gaissert H.A. (2021). Accuracy and Reproducibility of Intraoperative Assessment on Tumor Spread Through Air Spaces in Stage 1 Lung Adenocarcinomas. J. Thorac. Oncol..

[18] Metovic J., Falco E.C., Vissio E., Santoro F., Delsedime L., Massa F., Pittaro A., Osella-Abate S., Cassoni P., Volante M. (2021). Gross Specimen Handling Procedures Do Not Impact the Occurrence of Spread Through Air Spaces (STAS) in Lung Cancer. Am. J. Surg. Pathol..

[19] Xie H., Su H., Zhu E., Gu C., Zhao S., She Y., Ren Y., Xie D., Zheng H., Wu C. (2021). Morphological Subtypes of Tumor Spread Through Air Spaces in Non-Small Cell Lung Cancer: Prognostic Heterogeneity and Its Underlying Mechanism. Front. Oncol..

[20] Blaauwgeers H., Russell P.A., Jones K.D., Radonic T., Thunnissen E. (2018). Pulmonary loose tumor tissue fragments and spread through air spaces (STAS): Invasive pattern or artifact? A critical review. Lung Cancer.

[21] Myung J.K., Choe G., Chung D.H., Seo J.W., Jheon S., Lee C.T., Chung J.H. (2008). A simple inflation method for frozen section diagnosis of minute precancerous lesions of the lung. Lung Cancer.

[22] Blaauwgeers H., Flieder D., Warth A., Harms A., Monkhorst K., Witte B., Thunnissen E. (2017). A Prospective Study of Loose Tissue Fragments in Non-Small Cell Lung Cancer Resection Specimens: An Alternative View to “Spread Through Air Spaces”. Am. J. Surg. Pathol..

[23] Morimoto J., Nakajima T., Suzuki H., Nagato K., Iwata T., Yoshida S., Fukuyo M., Ota S., Nakatani Y., Yoshino I. (2016). Impact of free tumor clusters on prognosis after resection of pulmonary adenocarcinoma. J. Thorac. Cardiovasc. Surg..

[24] Cao H., Zheng Q., Deng C., Fu Z., Shen X., Jin Y., Yang Y., Qian B., Yuan C., Wang W. (2025). Prediction of spread through air spaces (STAS) by intraoperative frozen section for cT1N0M0 invasive lung adenocarcinoma: A multi-centre observational study (ECTOP-1016). Ann. Surg..

[25] Zhuo Y., Feng M., Yang S., Zhou L., Ge D., Lu S., Liu L., Shan F., Zhang Z. (2020). Radiomics nomograms of tumours and peritumoral regions for pre-operative prediction of spread through air spaces in lung adenocarcinoma. Transl. Oncol..

[26] Ma X., He W., Chen C., Tan F., Chen J., Yang L., Chen D., Xia L. (2025). A CT-based deep learning model for preoperative prediction of spread through air spaces in clinical stage I lung adenocarcinoma. Front. Oncol..

[27] Wang Y., Lyu D., Hu L., Wu J., Duan S., Zhou T., Tu W., Xiao Y., Fan L., Liu S. (2024). CT-based intratumoral and peritumoral radiomics nomograms for the preoperative prediction of spread through air spaces in clinical stage IA non-small cell lung cancer. J. Imaging Inform. Med..

[28] Feng Y., Ding H., Huang X., Zhang Y., Lu M., Zhang T., Wang H., Chen Y., Mao Q., Xia W. (2024). Deep learning-based detection and semi-quantitative model for spread through air spaces (STAS) in lung adenocarcinoma. npj Precis. Oncol..

[29] Wang S., Liu X., Jiang C., Kang W., Pan Y., Tang X., Luo Y., Gong J. (2024). CT-based super-resolution deep learning models with attention mechanisms for predicting spread through air spaces of solid or part-solid lung adenocarcinoma. Acad. Radiol..

[30] Tasnim S., Raja S., Mukhopadhyay S., Blackstone E.H., Toth A.J., Barron J.O., Raymond D.P., Bribriesco A.C., Schraufnagel D.P., Murthy S.C. (2024). Preoperative predictors of spread through air spaces in lung cancer: A narrative review. J. Thorac. Cardiovasc. Surg..

